# Community mental health under routine detention: jail reliance, pretrial custody, and frequent mental distress

**DOI:** 10.3389/fpubh.2026.1928427

**Published:** 2026-08-10

**Authors:** Xuanyin Li, Jinrui Zhang

**Affiliations:** 1 College of Criminal Justice, East China University of Political Science and Law, Shanghai, China; 2 Research Center for Public Security Governance, Hubei University of Police, Wuhan, Hubei, China

**Keywords:** community mental health, ecological study, frequent mental distress, jail incarceration, pretrial detention, public mental health, social determinants of health, U.S. counties

## Abstract

**Introduction:**

Most evidence on the criminal legal system as a structural determinant of population mental health concerns police killings and prison incarceration rather than the local jails and pretrial custody through which most criminal-legal contact occurs. We examined whether U.S. counties that rely more heavily on jail confinement and pretrial custody have a higher prevalence of frequent mental distress.

**Methods:**

We linked county-year frequent mental distress estimates from CDC PLACES (2018–2023) to jail population, pretrial custody, and jail admissions rates from the Vera Institute, law-enforcement incident rates from the FBI Crime Data Explorer, fatal police shooting rates from the Washington Post, and state-level jail mortality rates from the Bureau of Justice Statistics. Ecological associations were estimated with population-weighted least squares models with state and year fixed effects, county-clustered standard errors, and analysis windows matched to each source's coverage.

**Results:**

A one-standard-deviation higher jail population rate was associated with 1.17 percentage points higher frequent mental distress (95% CI 0.88 to 1.46; *n* = 6, 719). In 2018–2019, pretrial custody (*b* = 0.40) and jail admissions rates (*b* = 0.39) were independently associated with higher distress (*n* = 3, 289); law-enforcement incident (*b* = 0.47) and fatal police shooting rates (*b* = 0.25) showed smaller positive associations. After adjustment for county poverty, median income, racial and ethnic composition, population density, and urbanicity, all coefficients attenuated to near zero (jail population rate *b* = 0.06, 95% CI −0.03 to 0.15).

**Discussion:**

Counties that rely heavily on jail and pretrial detention carry a substantially higher community mental-health burden, but that burden is largely inseparable from the socioeconomic disadvantage of the same counties; routine detention practice is best treated as a marker of structurally disadvantaged community environments rather than as an isolated exposure.

## 1 Introduction

Mental distress is unevenly distributed across communities, and population mental health is increasingly studied as a product of institutional environments rather than of individual vulnerability alone (1, 2). Few institutions reach further into disadvantaged communities than the criminal legal system: more than three million people worldwide are held in pretrial or remand detention at any given moment (3), and each detention radiates outward into households, workplaces, and systems of care. Whether the routine operation of detention systems is visible in the mental health of whole communities, however, remains largely unmeasured, in part because the question requires small-area mental-health estimates and local custody data that few jurisdictions publish. The United States provides both: the CDC PLACES program estimates the county prevalence of frequent mental distress, defined as poor mental health on 14 or more of the previous 30 days (4), and county-administered jails generate unusually detailed public custody data. Research on structural racism and population health has used such place-level distress as a community condition through which institutional inequity can be observed and compared (5–8); within that tradition, this study asks whether communities that rely more heavily on routine detention also carry heavier mental-health burdens.

The strongest evidence to date concerns the system's most visible harms. Police killings damage the mental health of people far beyond those directly injured (9–12), and police contact, aggressive encounters, and neighborhood policing intensity have been linked to worse well-being and health across several populations, in the United States and beyond (13–18). A second line of research connects incarceration to psychiatric morbidity among residents of high-incarceration neighborhoods, to the well-being of family members, and to the health of children who witness a parent's arrest (19–23).

Most criminal-legal contact, however, occurs not through fatal encounters or prison sentences but through local jails and pretrial custody. Jails hold people awaiting trial, people serving short sentences, and people cycling through repeated admissions and releases, and they sit at the intersection of behavioral health systems, family networks, local courts, and policing (24–26). Pretrial detention imposes confinement before conviction and can disrupt care, employment, and household roles for defendants and their families (27–29). Because jails are administered by counties, county jail use is a marker of a local institutional pattern: how routinely a community relies on detention as a response to crisis, poverty, and legal processing (30–34). These routine processes rarely command the public attention that fatal police violence does, but they touch far more households.

There are concrete reasons to expect routine detention to register in community mental health. Each admission removes a resident from a household, and family members absorb caregiving gaps, lost earnings, and the strain of having a relative in custody (19–21). For people with mental-health or substance-use needs, booking interrupts treatment and medication continuity, and jails often substitute for scarce community services (23, 24, 35). Because jail stays are typically short and admissions frequent, these disruptions repeat across many households in high-admission counties, and pretrial custody adds unresolved legal status to the strain (22, 27, 28). The same processes can also run in reverse: communities with more untreated distress and weaker service systems may generate more detention (26, 30).

County-level research has begun to connect jail incarceration to population health: county jail incarceration rates predict county mortality (36, 37), jail incarceration rates are associated with more poor mental health days (25), and community behavioral-health infrastructure is related to jail burden (26, 30). Yet these strands remain separate. Studies of police violence, policing intensity, jail incarceration, and deaths in custody each examine one segment of the criminal-legal sequence, usually against different outcomes, samples, and periods. To our knowledge, no county-level study has estimated the associations of jail population, pretrial custody, jail admissions, law-enforcement incident rates, and fatal police shootings with the same community mental-health outcome. Without such a comparison, it is difficult to judge whether community mental distress is patterned mainly by the system's rare, highly visible events or also by its routine detention practices. That distinction matters for both legal epidemiology and community mental-health research, which treat law and legal institutions as population exposures (1, 2, 38–40).

Any such comparison must also confront the geography of disadvantage. Heavy jail use is concentrated where poverty is deep and services are thin, and the structural-racism and health literature argues that carceral exposure and socioeconomic deprivation are co-produced features of the same places, not independent axes that can be cleanly partialed apart (5–7). This entanglement cuts both ways: estimates that ignore county composition risk attributing to detention what belongs to poverty, while estimates that adjust for it risk discarding a pathway through which disadvantage operates (36). Rather than privileging either specification, this study reports unadjusted and covariate-adjusted estimates side by side and treats them as bounds.

This study addresses that gap using public county-year data for 2018–2023. We asked four questions. First, are county jail population rates associated with the prevalence of frequent mental distress? Second, are the procedural components of detention (pretrial custody and jail admissions) associated with distress independent of the overall jail population? Third, do broader law-enforcement incident rates show an association with distress beyond the jail measures? Fourth, do fatal police shooting rates, the exposure most studied in prior work, show a comparable association in the same framework? For each question, we then examined whether the association persists after adjustment for county socioeconomic and demographic composition. We also examined state-level jail mortality as a supplementary analysis, because public mortality data are not released at the county level.

The design is ecological and associational. County detention practice is plausibly entangled with the same structural disadvantage that shapes mental health, the data contain no discrete policy change to exploit, and the models compare county-year environments instead of following individuals. The contribution is therefore descriptive: to estimate, within one framework and with analysis windows matched to what each public data source can support, which parts of the criminal-legal sequence are associated with community mental distress, and how much of that association is separable from county disadvantage.

## 2 Materials and methods

### 2.1 Study design

We conducted an ecological study of U.S. county-years. Counties are the relevant administrative unit because jails, pretrial practices, and many court-linked detention decisions are organized at the county level, and because the closest prior work on jail incarceration and population health is county-based (25, 26, 36). Coefficients from this design describe differences in population-level distress across county-year environments; they do not describe changes in any individual's mental health following criminal-legal contact.

Because the exposure sources differ in coverage, the analysis uses separate windows matched to each source instead of a single pooled panel: 2018–2023 for the jail population measures, 2018–2019 for pretrial custody and admissions (the years in which these measures are most completely observed, before pandemic-era disruptions to jail operations and reporting), 2021–2023 in a 20-state sub-sample for law-enforcement incident data, 2018–2023 for fatal police shootings, and 2018–2019 at the state level for jail mortality. Table 1 summarizes the sources, measures, and windows. The study is reported in accordance with the STROBE (Strengthening the Reporting of Observational Studies in Epidemiology) recommendations for cross-sectional studies [(41); Supplementary Data Sheet 1].

**Table 1:** Data sources, measures, and analysis windows.

Source	Measures	Window and scope	Role in the analysis
CDC PLACES	Frequent mental distress (%)	County estimates, 2018–2023	Outcome in all analyses; modeled small-area estimate.
Vera incarceration trends	Jail population rate; pretrial custody rate; jail admissions rate; pretrial share of jail; capacity utilization	2018–2023 (jail population); 2018–2019 (pretrial, admissions)	Primary exposures.
FBI crime data explorer (LEARCAT)	Total incident rate; drug incident rate	2021–2023, 20-state sub-sample with stable reporting	Secondary exposure; reported incidents, not arrests.
Washington post fatal force	Fatal police shootings rate; any-shooting indicator (sensitivity)	2018–2023 county-year panel	Secondary exposure linking to the police-violence literature.
Census SAIPE; vera demographics	Poverty rate; median household income; percent Black; percent Hispanic/Latino; population density; urbanicity	2018–2023, county-year	Covariates in adjusted models.
BJS local-jail mortality	State-year jail mortality rate	2018–2019, state level	Supplementary analysis; not released at the county level.

All county analyses share the PLACES outcome and the Vera jail measures. Exposures are standardized within each analytic sample.

### 2.2 Data sources and measures

The outcome is county frequent mental distress from CDC PLACES: the model-based estimated percentage of adults reporting poor mental health on 14 or more of the previous 30 days (4). PLACES provides small-area estimates, not direct county surveys, so the outcome is a modeled population-level prevalence rather than an aggregate of raw responses; the implications of this construction for the covariate-adjusted models are discussed below.

Detention measures come from the Vera Institute's Incarceration Trends county panel (42). The jail population rate is the average daily jail population per 100,000 residents ages 15–64; it measures the standing level of county reliance on jail confinement. The pretrial custody rate is the average daily pretrial population per 100,000 residents ages 15–64; we computed it directly from the reported pretrial custody counts and population denominators, because the precomputed pretrial rate field in the source file is not scaled consistently with the jail population rate field. The jail admissions rate (admissions per 100,000 residents ages 15–64) measures how many people enter jail during the year; admissions capture turnover that a standing population rate can miss, because frequent short stays can disrupt many households even when the average daily population is moderate (22, 24, 27). Two derived measures describe the composition and intensity of confinement: the pretrial share of the jail population, and capacity utilization (jail population divided by rated capacity).

Three additional sources extend the comparison. Law-enforcement incident rates (total incidents and drug/narcotic incidents per 100,000 residents) come from the FBI Crime Data Explorer county-level LEARCAT extracts (43); these measure reported incidents, not arrests or charges. Fatal police shooting rates come from the Washington Post Fatal Force database (44). State-year jail mortality rates come from the Bureau of Justice Statistics (BJS) mortality tables (45); the public release does not include county-level custody deaths, so this measure enters only a supplementary state-level analysis.

Covariates for the adjusted models describe county socioeconomic and demographic composition: the county poverty rate and log median household income from the Census Small Area Income and Poverty Estimates (SAIPE), the percentages of residents ages 15–64 who are Black and who are Hispanic/Latino and the urbanicity category from the Vera panel, and log population density.

### 2.3 Analytic samples

Primary analytic samples for the jail, pretrial, and fatal-force models exclude county-years with fewer than 10,000 residents ages 15–64 and counties served by regional (multi-county) jails, because small denominators and shared facilities distort county rates; the incident analysis instead uses all county-years in 20 states with stable incident reporting in 2021–2023. Table 2 traces each primary sample from the linked county-years through these exclusions to the complete-case observations used in estimation.

**Table 2:** Construction of the primary analytic samples.

Model (headline exposures)	Window and scope	Linked county-years (counties)	After exclusions	Primary model *n*
Model 1: jail population	2018–2023, national	14,818 (3,054)	8,909	6,719
Model 2: pretrial custody, admissions	2018–2019, national	6,091 (3,053)	3,535	3,289
Model 3: incident rates	2021–2023, 20 states	3,684 (1,316)	—	2,748
Model 4: fatal shootings	2018–2023, national	14,818 (3,054)	8,909	7,046

Linked county-years have the PLACES outcome and Vera jail measures in the window (for Model 3, all county-years in the 20 states with stable incident reporting). Exclusions drop county-years with fewer than 10,000 residents ages 15–64 and counties served by regional jails; the primary incident model applies no such exclusions, while its stricter sensitivity sample does and additionally requires at least 90% NIBRS population coverage (*n* = 1, 293). Primary model *n* are complete cases on the variables of the corresponding model in Table 4. The supplementary state-level mortality analysis uses 89 state-years.

The step from the exclusion-eligible county-years to the complete cases is driven almost entirely by missingness in the Vera confinement-detail fields rather than in the outcome: among exclusion-eligible county-years in 2018–2023, pretrial custody counts and rated jail capacity are each missing for about 19% of county-years and admissions for about 29%, whereas the distress outcome is missing for 2% and the jail population rate for under 1%. The same fields are far more complete in 2018–2019 (about 6% and 3% missing for pretrial custody and admissions), consistent with the choice of that window for the procedural measures. County-years lost to missingness are smaller (median population ages 15–64 of about 28,000, vs. 42,000 among complete cases) and more often rural (57% vs. 42%), with slightly higher average distress (16.1% vs. 15.8%), so the primary samples somewhat under-represent small rural counties with high distress. We use complete-case estimation without imputation; the less restricted sensitivity samples reported alongside each primary model relax the population and regional-jail exclusions and show how the estimates change when more county-years enter.

### 2.4 Statistical analysis

All models estimate same-year associations between standardized exposures and the frequent mental distress percentage:


$$\begin{array}{l} Y_{ct}=\alpha +\underset{k}{\sum }\beta_{k}Z_{ct}^{\left(k\right)}+\gamma_{s\left(c\right)}+\delta_{t}+\varepsilon_{ct}, \end{array}$$ (1)

where *Y*_*ct*_ is frequent mental distress in county *c* and year *t*, $Z_{ct}^{\left(k\right)}$ are exposures standardized (*z*-scored) within each analytic sample, and γ_*s*(*c*)_ and δ_*t*_ are state and year fixed effects. Models are estimated by weighted least squares with county total population as weights. Weighting makes the estimates person-representative rather than county-representative: they describe the county environment experienced by the average resident, and they prevent the thousands of small counties, where both the outcome and the exposures are measured least reliably, from dominating the fit. The state fixed effects absorb stable between-state differences in policy regimes, court organization, and reporting systems; the year fixed effects absorb national shocks, including pandemic-era disruptions to jail operations. The coefficients are therefore identified from cross-county variation within states. Standard errors are clustered by county to account for repeated observation of the same county across years; heteroskedasticity-robust (HC3) standard errors yield similar conclusions. Each β_*k*_ is the percentage-point difference in distress associated with a one-standard-deviation difference in the exposure. The supplementary state-level model regresses state distress on state jail population, pretrial custody, and jail mortality rates with year fixed effects, weighted by state population, with standard errors clustered by state. All analyses were conducted in Python using the open-source pandas and statsmodels libraries.

Four primary county models were estimated: (1) jail population rate, pretrial share of jail, and capacity utilization, 2018–2023; (2) jail population, pretrial custody, and jail admissions rates, 2018–2019; (3) jail population, pretrial custody, total incident, and drug incident rates in the 20-state sub-sample, 2021–2023; and (4) jail population, pretrial custody, and fatal police shooting rates, 2018–2023. For each data source, one primary specification was selected before the manuscript was drafted, on the basis of the source's coverage and measurement properties, not the size of estimated coefficients. Four alternative specifications are reported as sensitivity analyses: less restricted samples, a two-exposure state-fixed-effects model over the full window, a binary any-shooting indicator, and a first-difference model.

To assess confounding by county socioeconomic composition, each primary model was re-estimated adding the socioeconomic and demographic covariates described above. Two caveats shape the interpretation of these adjusted models. First, PLACES small-area estimates are partly model-based on county sociodemographic predictors, so socioeconomic covariates can absorb outcome variation mechanically, over-attenuating true associations. Second, detention burden may itself be one pathway through which disadvantage operates, in which case adjustment removes part of the association of interest. We therefore interpret the unadjusted and adjusted estimates as bounds on the detention–distress association rather than treating either as a causal parameter.

## 3 Results

### 3.1 Descriptive patterns

Counties with heavier detention burden report more frequent mental distress (Table 3). In 2018–2019, counties in the lowest jail-population-rate quartile (mean 167 per 100,000) averaged 13.3% frequent mental distress vs. 16.1% in the highest quartile (mean 1,383 per 100,000). The gradient for pretrial custody rates ran from 13.6% in the lowest quartile (mean 81 per 100,000) to 15.8% in the highest (mean 720 per 100,000). In the 2018–2023 window, the jail-population contrast was 14.6% vs. 16.9%. The pretrial share of jail shows no such gradient (15.8% vs. 15.5%), consistent with its nature as a compositional measure: a county can have a high pretrial share because it detains many people before trial, detains few sentenced people, or has a small jail overall. These contrasts are unadjusted; they motivate the models but do not account for confounding.

**Table 3:** Frequent mental distress by detention exposure quartiles.

Window	Exposure	Q1 mean exposure	Q4 mean exposure	Q1 mean FMD	Q4 mean FMD
2018–2019	Jail population rate	167	1,383	13.3%	16.1%
2018–2019	Pretrial custody rate	81	720	13.6%	15.8%
2018–2023	Jail population rate	150	1,408	14.6%	16.9%
2018–2023	Pretrial share of jail	0.32	0.91	15.8%	15.5%

FMD, frequent mental distress (CDC PLACES). Rates are per 100,000 residents ages 15–64. Quartile means are computed in the analytic samples and are not adjusted for covariates or fixed effects.

### 3.2 Jail population rate, 2018–2023

**Figure 1:**
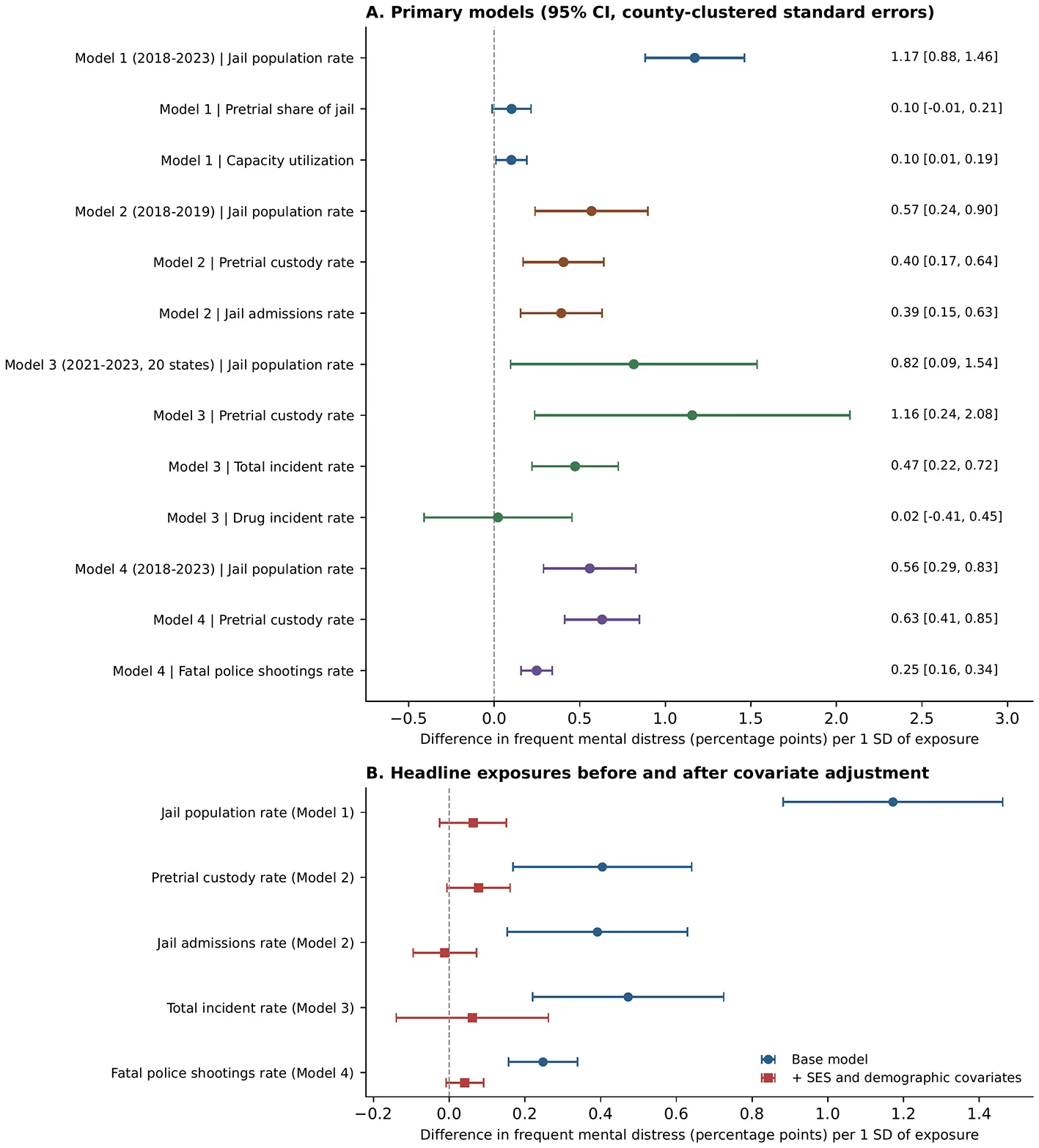
Coefficient estimates from the primary and covariate-adjusted models. **(A)** Shows standardized coefficients with 95% confidence intervals (county-clustered standard errors) from the four primary county-level models in Table 4. **(B)** Compares the headline exposure in each model before and after adjustment for county poverty, median income, racial and ethnic composition, population density, and urbanicity (Table 5).

In the primary 2018–2023 model (Table 4, Model 1), a one-standard-deviation higher jail population rate was associated with 1.17 percentage points higher frequent mental distress (95% CI 0.88 to 1.46, *p* < 0.001; *n* = 6, 719). Coefficients from all four primary models are displayed in Figure 1A. The pretrial share of jail (0.10, 95% CI −0.01 to 0.21, *p* = 0.081) and capacity utilization (0.10, 95% CI 0.01 to 0.19, *p* = 0.031) showed small positive associations near the margin of statistical significance. In a less restricted sample without the population and regional-jail exclusions, the jail-population estimate was similar (1.29, 95% CI 0.85 to 1.72; *n* = 10, 954). A first-difference model relating year-to-year changes in jail population to changes in distress was null, so the association should be interpreted as a cross-county pattern rather than as evidence that short-run within-county changes in jail population change distress.

**Table 4:** Primary county-level models of frequent mental distress.

Exposure or model detail	Model 1	Model 2	Model 3	Model 4
Jail population rate (z)	1.17 [0.88, 1.46]	0.57 [0.24, 0.90]	0.82 [0.09, 1.54]	0.56 [0.29, 0.83]
Pretrial custody rate (z)		0.40 [0.17, 0.64]	1.16 [0.24, 2.08]	0.63 [0.41, 0.85]
Jail admissions rate (z)		0.39 [0.15, 0.63]		
Pretrial share of jail (z)	0.10 [−0.01, 0.21]			
Capacity utilization (z)	0.10 [0.01, 0.19]			
Total incident rate (z)			0.47 [0.22, 0.72]	
Drug incident rate (z)			0.02 [−0.41, 0.45]	
Fatal shootings rate (z)				0.25 [0.16, 0.34]
Window	2018–2023	2018–2019	2021–2023	2018–2023
Scope	National	National	20-state sub-sample	National
Observations	6,719	3,289	2,748	7,046
*R* ^2^	0.628	0.571	0.488	0.634

Entries are coefficients with 95% confidence intervals in brackets, from population-weighted least squares models with state and year fixed effects and standard errors clustered by county. Exposures are standardized within each analytic sample; the outcome is the frequent mental distress percentage. Model 3 is restricted to a 20-state sub-sample with stable incident reporting and is not nationally representative.

The magnitude of the unadjusted jail-population association is substantial in a population-health frame. Mean frequent mental distress across the analytic county-years is about 15.7%, so 1.17 percentage points amounts to roughly a 7% relative difference in prevalence per standard deviation of jail population rate, and to about half of the unadjusted gap between the lowest and highest jail-population quartiles in Table 3 (14.6% vs. 16.9%). In a county with 100,000 adults, the same difference corresponds to roughly 1,200 additional adults reporting frequent mental distress. The estimate is ecological and should not be translated into an individual-level risk.

### 3.3 Pretrial custody and jail admissions, 2018–2019

In the primary 2018–2019 model (Model 2), jail population rate (0.57, 95% CI 0.24 to 0.90, *p* < 0.001), pretrial custody rate (0.40, 95% CI 0.17 to 0.64, *p* < 0.001), and jail admissions rate (0.39, 95% CI 0.15 to 0.63, *p* = 0.001) were each positively associated with frequent mental distress (*n* = 3, 289). Counties where more people were held before trial and where more people entered jail during the year thus reported higher distress, over and above the standing jail population.

The pattern held in less restricted samples. Without the population and regional-jail exclusions, the pretrial custody (0.46, 95% CI 0.05 to 0.86, *p* = 0.026) and admissions (0.66, 95% CI 0.39 to 0.93, *p* < 0.001) associations remained positive (*n* = 5, 432). In a two-exposure specification over the full 2018–2023 window (*n* = 11, 483), where pretrial custody is less completely observed, the pretrial custody association was also positive (1.09, 95% CI 0.69 to 1.50, *p* < 0.001).

### 3.4 Law-enforcement incident rates, 2021–2023

In the 20-state sub-sample (Model 3), a one-standard-deviation higher total incident rate was associated with 0.47 percentage points higher distress (95% CI 0.22 to 0.72, *p* < 0.001; *n* = 2, 748), in a model that also included jail population and pretrial custody rates, both of which remained positive. The drug-specific incident rate showed no association (0.02, 95% CI −0.41 to 0.45, *p* = 0.92). In the stricter sensitivity sample with the population, regional-jail, and NIBRS-coverage restrictions (*n* = 1, 293), the total incident association was similar (0.41, 95% CI 0.23 to 0.58). The incident measure reflects reported incidents rather than arrests or charges, and it cannot distinguish crime prevalence from reporting and enforcement practice; the sub-sample is also not nationally representative. The result therefore indicates only that a broader measure of the local criminal-legal environment is associated with distress where reporting is stable, not that drug enforcement specifically drives the pattern.

### 3.5 Fatal police shootings, 2018–2023

A one-standard-deviation higher fatal police shooting rate was associated with 0.25 percentage points higher distress (95% CI 0.16 to 0.34, *p* < 0.001; *n* = 7, 046; Model 4), with the jail population (0.56, 95% CI 0.29 to 0.83) and pretrial custody (0.63, 95% CI 0.41 to 0.85) associations remaining positive in the same model. This is consistent with the police-killing spillover literature (9–12), and it indicates that the detention associations do not simply proxy for fatal police violence.

In a sensitivity specification using a binary indicator for any fatal shooting in a county-year (*n* = 11, 483), the coefficient was negative (−0.26, 95% CI −0.42 to −0.10). Whether any shooting occurs is strongly related to county population size, so the binary indicator compares compositionally different counties instead of measuring exposure intensity; we interpret the reversal as a compositional artifact and treat the rate specification as primary.

### 3.6 Socioeconomic and demographic adjustment

Adjustment for county socioeconomic composition attenuated all of the criminal-legal associations to near zero (Table 5; Figure 1B). With county poverty rate, log median household income, percent Black, percent Hispanic/Latino, log population density, and urbanicity added, the jail population coefficient in Model 1 fell from 1.17 to 0.06 (95% CI −0.03 to 0.15), the pretrial custody coefficient in Model 2 fell from 0.40 to 0.08 (95% CI −0.01 to 0.16), the admissions coefficient fell to −0.01, the total incident coefficient fell to 0.06, and the fatal shooting coefficient fell to 0.04 (95% CI −0.01 to 0.09). Model *R*^2^ rose from 0.49–0.63 to 0.77–0.87, with log median income (about −1.0 to −1.1 percentage points per standard deviation) and poverty (about +0.4) the dominant covariates.

**Table 5:** Covariate-adjusted models of frequent mental distress.

Exposure or model detail	Model 1	Model 2	Model 3	Model 4
Jail population rate (z)	0.06 [−0.03, 0.15]	0.04 [−0.06, 0.14]	0.00 [−0.21, 0.21]	0.01 [−0.09, 0.11]
Pretrial custody rate (z)		0.08 [−0.01, 0.16]	0.05 [−0.21, 0.30]	0.07 [−0.02, 0.16]
Jail admissions rate (z)		−0.01 [−0.10, 0.07]		
Total incident rate (z)			0.06 [−0.14, 0.26]	
Fatal shootings rate (z)				0.04 [−0.01, 0.09]
Observations	6,719	3,289	2,748	7,046
*R* ^2^	0.844	0.871	0.765	0.842

Each column re-estimates the corresponding primary model in Table 4 adding county poverty rate, log median household income (Census SAIPE), percent Black and percent Hispanic/Latino residents ages 15–64, log population density, and urbanicity category. Entries are coefficients with 95% confidence intervals in brackets; standard errors are clustered by county. Model 1 also retains pretrial share of jail and capacity utilization; Model 3 retains the drug incident rate (coefficients not shown, all near zero).

Adjustment therefore removes roughly 80% or more of every unadjusted coefficient. The adjusted intervals still admit small positive associations (upper bounds of about 0.15 percentage points per standard deviation for the jail population rate and 0.16 for pretrial custody), but they exclude anything close to the unadjusted magnitudes. What the adjusted models establish is that the detention–distress association is not separable from county socioeconomic disadvantage in cross-sectional public data; whether the attenuation reflects confounding, over-adjustment of a mediated pathway, mechanical absorption arising from the PLACES outcome construction, or some mixture is taken up in the Discussion.

### 3.7 State-level jail mortality, 2018–2019

In the supplementary state-level analysis (*n* = 89 state-years), the jail mortality rate was positively but imprecisely associated with distress (0.25, 95% CI −0.21 to 0.72, *p* = 0.29); the estimate is not statistically significant. County-level versions of this analysis, constructed by assigning state mortality rates to counties, were unstable across specifications. Because the public BJS release does not report county custody deaths, these data cannot support county-level conclusions, and we report the state-level estimate for completeness only.

## 4 Discussion

This ecological study of U.S. counties yields two principal findings. First, community mental distress is robustly associated with the routine components of local detention in unadjusted models: the jail population rate showed the largest association in the 2018–2023 window, pretrial custody and jail admissions rates were independently associated with distress in 2018–2019, and these associations were at least as large as those for law-enforcement incident rates and fatal police shootings estimated in the same framework. Second, all of these associations attenuated to near zero once county poverty, income, demographic composition, and urbanicity were taken into account. The sections below consider what routine detention adds as a population mental-health exposure, how the attenuation should be interpreted, and why the pattern matters beyond the country in which it was estimated.

### 4.1 Routine detention as a population mental-health exposure

The unadjusted results give empirical standing to the least visible part of the criminal-legal sequence. Prior county-level work had linked jail incarceration rates to poor mental health days (25) and to county mortality (36, 37); this study extends that work by using a defined mental-health prevalence outcome, separating the standing jail population from its procedural components, and benchmarking detention against policing exposures within one framework. The comparison matters because research attention has concentrated on the system's rare, highly visible harms. The fatal-shooting association replicates the direction of the police-killing spillover literature (9, 11, 12), but the detention measures retained unadjusted associations that were at least as large, which suggests that community mental distress is patterned by what detention systems do routinely and not only by their most publicized events.

The secondary layers sharpen this reading. Where incident reporting is stable, distress co-varies with a broad measure of the local criminal-legal environment, yet the jail and pretrial terms survive alongside it, and the null for drug-specific incidents warns against reducing that environment to any single enforcement practice; the detention associations are neither a proxy for fatal police violence nor a shadow of generic crime exposure. The framework's boundary is equally telling. Deaths in custody, the most severe outcome a detention system can produce, could be examined only at the state level, where the estimate was positive but imprecise, because no public release connects custody deaths to counties. The criminal-legal sequence is thus easiest to measure where it is most routine and hardest to measure where it is most lethal. An account that rests on what is conveniently observable will therefore understate the system's severest harms.

If any part of the unadjusted association is causal, the mechanisms sketched in the Introduction indicate where to look, and the results discriminate among them to a degree. The independent associations of admissions and pretrial custody are what a disruption account predicts: repeated short stays and unresolved legal status spread acute strain across many households (27–29), interrupt treatment for people whose care already runs through fragile systems (23, 24, 35, 46, 47), and tax the family members who absorb caregiving gaps and lost earnings (19–21). A pure stock account, in which only the standing size of the jail population mattered, would not predict those procedural coefficients. Reverse pathways remain equally plausible: communities with higher distress and weaker service systems may generate more detention (26, 30).

### 4.2 Detention burden and the geography of disadvantage

The attenuation under socioeconomic adjustment admits three readings that these data cannot separate: confounding by county disadvantage, over-adjustment of a pathway through which disadvantage operates, and a mechanical component arising because the PLACES outcome is itself partly constructed from sociodemographic predictors. The unadjusted and adjusted estimates are therefore best read as bounds. Within those bounds, the attenuation should not be taken to show that detention is irrelevant to community mental health; what it establishes is that, in cross-sectional public data, routine detention burden marks county environments with substantially worse community mental health, and this marking operates through, not apart from, the broader geography of disadvantage.

That inseparability is itself the equity finding, and it echoes the core claim of the structural-racism and health literature: carceral exposure and socioeconomic deprivation are not separate axes of disadvantage but jointly concentrated features of the same places (5–7, 48). The places where detention is heaviest are the places where poverty is deepest and distress is highest, and criminal-legal contact, detention, and health vulnerability are unevenly distributed across race, class, and disability (49–51). A county-level analysis cannot show who within a county bears this burden, and the results should not be read as an individual-level stratification analysis; they complement, rather than replace, individual and neighborhood studies of family incarceration, police contact, and racialized health inequity (13, 15, 16, 52).

### 4.3 Routine detention in global perspective

Although estimated in U.S. counties, the exposure studied here is the modal form of criminal-legal confinement worldwide. More than three million people are held in pretrial or remand detention at any given time, roughly one third of the global prison population, and their number has grown faster than the world's population since 2000 (3, 53). In about half the countries of Africa and South Asia, more than 40% of prisoners are awaiting trial; remand detainees form the majority of the prison population in countries as different as India, Bangladesh, Nigeria, the Philippines, Bolivia, and Paraguay; and pretrial detention is a leading driver of prison overcrowding in Latin America (3, 53). Routine custody is thus most common in regions where it often coincides with much thinner community mental-health service infrastructure (54) and where detention intersects directly with poverty.

The United States is, in this respect, less an exceptional case than an unusually well-documented one: few other systems publish facility-level custody counts alongside small-area mental-health estimates, and it is this documentation that makes the coupling between routine detention and community mental health estimable at all. What is most likely to travel is not the coefficients, which reflect distinctive institutions such as county-administered jails and cash bail, but the coupling itself: where detention is routine, its community mental-health burden is embedded in, and marks, broader structural disadvantage, so that mental-health planning and detention policy concern the same places. In jurisdictions with higher remand shares and thinner service systems, routine custody may be an even more informative marker of community mental-health risk, a hypothesis this study cannot test but that its design can be adapted to examine; individual-level evidence that criminal-legal contact damages mental health already extends well beyond the United States (18), and what is missing is the community-level surveillance layer. The methodological template is portable, and it is legal epidemiology in the strict sense of treating law and legal institutions as population exposures (38, 40): link small-area mental-health estimates to administrative custody data, match analysis windows to what each source can support, and bound rather than assert causal claims. Applying it will require the subnational custody data (including deaths in custody) and small-area health estimates that most countries do not yet publish, which is itself a data-infrastructure implication of this study.

## 5 Conclusion

Three limits define the scope of these findings, and each points to where the next studies should go. First, the design is ecological and cross-sectional: it compares county-year environments and cannot establish whether detention burden raises distress, distress generates detention, or both express shared structural conditions. The way forward is variation over time and policy: bail and pretrial reforms, decarceration initiatives, and jail closures create quasi-experimental leverage that county comparisons lack, ideally combined with individually linked longitudinal data. Second, the measurement apparatus imposes interpretive ceilings: the PLACES outcome is a modeled small-area estimate whose uncertainty the regressions do not propagate, and socioeconomic adjustment removes mediated pathways along with confounding, which is why we report bounds rather than a point estimate. Progress here means more directly measured outcomes and designs built to decompose the disadvantage–detention–distress pathway rather than to partial it away. Third, public data thin out exactly where the questions sharpen: pretrial and admissions measures are well observed only in 2018–2019 and required recomputation from raw counts, incident data support a 20-state sub-sample, and custody deaths are not released below the state level. These gaps are specific, fixable reporting decisions; county-level releases of pretrial, admissions, and custody-death data would advance this literature more than any statistical remedy, in the United States and in the many jurisdictions where remand detention is even more prevalent.

Within those bounds, the evidence supports a clear conclusion. Counties that rely more heavily on jail confinement and pretrial custody report substantially worse community mental health, and these unadjusted associations are at least as strong as those for the criminal-legal exposures that have received the most research attention; that burden, however, is largely inseparable from county socioeconomic disadvantage in cross-sectional data. Routine detention practice is therefore best understood as part of the institutional fabric of disadvantaged community environments—a marker worth monitoring in community mental-health research and a candidate pathway for intervention—rather than as an isolated exposure.

## Data Availability

Publicly available datasets were analyzed in this study. These data can be found at: CDC PLACES (https://www.cdc.gov/places/), Vera Institute of Justice Incarceration Trends (https://github.com/vera-institute/incarceration-trends), FBI Crime Data Explorer (https://cde.ucr.cjis.gov/LATEST/webapp/), the Washington Post Fatal Force database (https://www.washingtonpost.com/graphics/investigations/police-shootings-database/), Bureau of Justice Statistics Mortality in Local Jails (https://bjs.ojp.gov/library/publications/mortality-local-jails-2000-2019-statistical-tables), and U.S. Census Bureau SAIPE (https://www.census.gov/programs-surveys/saipe.html).

## Supplementary material

Supplementary Data Sheet 1 Strobe checklist for the reporting of cross-sectional studies, completed for this article.
