# Supplementary material for: Community mental health under routine detention: jail reliance, pretrial custody, and frequent mental distress
Source: Front Public Health. 2026 Aug 10;14:1928427. doi: 10.3389/fpubh.2026.1928427 (PMC13500246; doi:10.3389/fpubh.2026.1928427)
Supplement: Supplementary file 1 [file Data_Sheet_1.pdf]

STROBE Statement — Checklist of items for cross-sectional studies

Manuscript: *Community Mental Health Under Routine Detention: Jail Reliance, Pretrial Custody, and Frequent Mental Distress* (Frontiers in Public Health, Original Research). Section and table numbers refer to the submitted manuscript.

Item	No.	Recommendation	Location in manuscript
Title and abstract	1	(a) Indicate the study's design with a commonly used term in the title or the abstract. (b) Provide an informative and balanced summary of what was done and what was found.	Abstract (structured; "ecological associations" stated in Methods sentence; findings and attenuation both reported)
Background/rationale	2	Explain the scientific background and rationale for the investigation being reported.	Introduction, paragraphs 1–6
Objectives	3	State specific objectives, including any prespecified hypotheses.	Introduction, research-questions paragraph (four questions plus adjustment)
Study design	4	Present key elements of study design early in the paper.	Section 2.1 (ecological cross-sectional design; window-matched panels)
Setting	5	Describe the setting, locations, and relevant dates, including periods of recruitment, exposure, follow-up, and data collection.	Section 2.1 and Table 1 (U.S. county-years; source-specific windows, 2018–2023)
Participants	6	(a) Give the eligibility criteria, and the sources and methods of selection of participants.	Section 2.3 and Table 2 (population and regional-jail exclusions; linked county-years to complete cases)
Variables	7	Clearly define all outcomes, exposures, predictors, potential confounders, and effect modifiers.	Section 2.2 and Table 1 (outcome, five exposure layers, derived measures, covariates)
Data sources/ measurement	8	For each variable of interest, give sources of data and details of methods of assessment (measurement).	Section 2.2 (including recomputation of the pretrial custody rate from raw counts)
Bias	9	Describe any efforts to address potential sources of bias.	Sections 2.2 and 2.4 (PLACES construction caveats; population weighting; state and year fixed effects; clustered standard errors); Sections 3.5 and 4.2 (compositional artifact; bounds interpretation)

Item	No.	Recommendation	Location in manuscript
Study size	10	Explain how the study size was arrived at.	Section 2.3 and Table 2 (sample-construction flow for each primary model)
Quantitative variables	11	Explain how quantitative variables were handled in the analyses.	Section 2.4 (exposures standardized within analytic samples; outcome in percentage points)
Statistical methods	12	(a) Describe all statistical methods, including those used to control for confounding. (b) Describe any methods used to examine subgroups and interactions. (c) Explain how missing data were addressed. (d) If applicable, describe analytical methods taking account of sampling strategy. (e) Describe any sensitivity analyses.	Section 2.4 (weighted least squares, fixed effects, clustered standard errors, covariate adjustment); Section 2.3 (complete-case estimation, missingness analysis); Sections 2.4 and 3.2–3.5 (sensitivity analyses)
Participants (results)	13	Report numbers of individuals at each stage of the study.	Table 2; observation counts in Tables 4 and 5
Descriptive data	14	(a) Give characteristics of study participants and information on exposures and potential confounders. (b) Indicate number of participants with missing data for each variable of interest.	Section 3.1 and Table 3 (exposure quartiles); Section 2.3 (variable-level missingness and profile of excluded county-years)
Outcome data	15	Report numbers of outcome events or summary measures.	Sections 3.1–3.7; Tables 3–5
Main results	16	(a) Give unadjusted estimates and, if applicable, confounder-adjusted estimates and their precision (e.g., 95% confidence intervals). (b) Report category boundaries when continuous variables were categorized. (c) If relevant, consider translating estimates of relative risk into absolute risk.	Tables 4 and 5 (unadjusted and adjusted coefficients with 95% CIs); Table 3 (quartile boundaries); Section 3.2 (absolute interpretation: percentage points, affected adults per 100,000)
Other analyses	17	Report other analyses done (e.g., analyses of subgroups and interactions, and sensitivity analyses).	Sections 3.2–3.5 (less restricted samples, binary coding, first-difference model); Section 3.7 (state-level mortality)
Key results	18	Summarise key results with reference to study objectives.	Section 4, opening paragraph; Section 5
Limitations	19	Discuss limitations of the study, taking into account sources of potential bias or imprecision.	Section 5, first paragraph; Sections 2.2 and 2.4 (measurement and adjustment caveats)
Interpretation	20	Give a cautious overall interpretation of results considering objectives, limitations, multiplicity of analyses, results from similar studies, and other relevant evidence.	Sections 4.1–4.3 and 5 (bounds framing; comparison with prior county-level and police-violence literatures)

Item	No.	Recommendation	Location in manuscript
Generalisability	21	Discuss the generalisability (external validity) of the study results.	Section 4.3 (scope of transportability beyond the United States); Section 2.3 (restricted-sample caveat)
Funding	22	Give the source of funding and the role of the funders for the present study.	Funding statement
